# Robot-Assisted Thoracoscopic Surgery (RATS) in the Prone Position for a Posterior Mediastinal Tumor

**DOI:** 10.7759/cureus.82895

**Published:** 2025-04-24

**Authors:** Hideki Motoyama, Taishi Adachi, Takao Nakanishi, Momoko Soda, Mitsugu Omasa

**Affiliations:** 1 Department of Thoracic Surgery, Kobe City Nishi-Kobe Medical Center, Kobe, JPN

**Keywords:** adamkiewicz artery, posterior mediastinal tumor, prone position, robot-assisted thoracoscopic surgery, schwannoma

## Abstract

Posterior mediastinal tumors are typically addressed using video-assisted thoracoscopic surgery (VATS) or robot-assisted thoracoscopic surgery (RATS), both of which are usually performed with the patient in the lateral decubitus position. However, tumors in confined spaces require optimal surgical visualization and maneuverability. We report the successful resection of a schwannoma located between the thoracic vertebrae and the descending aorta using robot-assisted thoracoscopic surgery in the prone position. A 67-year-old man presented with a left posterior mediastinal tumor located between the sixth and eighth vertebrae and the descending aorta. Due to the tumor's proximity to critical structures, we opted for robot-assisted thoracoscopic surgery in the prone position to enhance visualization and maneuverability in the confined space. Following general anesthesia, the patient was placed in the prone position, and three robotic ports, along with one assist port, were inserted into the left thoracic cavity. The tumor was carefully dissected using robotics, with continuous monitoring of its surface, the aorta, and the intercostal arterial branch. The procedure was completed without complications, and the patient was discharged on postoperative day 4. This case highlights the feasibility and safety of robot-assisted thoracoscopic surgery in the prone position for the resection of posterior mediastinal tumors, particularly those in confined anatomical locations. This approach offers superior visualization and maneuverability, reducing the risk of intercostal artery injury and potential complications related to the Adamkiewicz artery.

## Introduction

Video-assisted thoracoscopic surgeries (VATS) and robot-assisted thoracoscopic surgeries (RATS) are typically performed in the lateral decubitus position for posterior mediastinal tumors [1]. However, tumors adjacent to the inferior thoracic vertebral body require special consideration due to the high likelihood that the Adamkiewicz artery originates from the dorsal branch of the left intercostal artery at the level of the seventh to 12th thoracic vertebrae as demonstrated by Kieffer et al. for more than half of 419 Adamkiewicz arteries [2]. The Adamkiewicz artery flows into the anterior spinal artery and supplies the anterior two-thirds of the spinal cord. A reduction in blood flow through this artery can lead to spinal cord ischemia, potentially resulting in paraplegia. Therefore, careful attention is required during surgical procedures involving this vessel [3]. Additionally, tumors in contact with the aorta are near the intercostal artery's origin, making hemorrhage particularly challenging to control. Therefore, optimal surgical visualization and maneuverability are crucial. The prone position provides a clear surgical field by utilizing gravity to compress the lungs, and the robotic instruments, with their articulated joints, allow precise manipulation to avoid the aorta. Here, we report the successful resection of a schwannoma located between the left side of the sixth to eighth thoracic vertebrae and the descending aorta using robot-assisted thoracoscopic surgery in the prone position. This approach provided superior visualization and maneuverability, minimizing the risk of intercostal arterial injury.

## Case presentation

**Figure 1:**
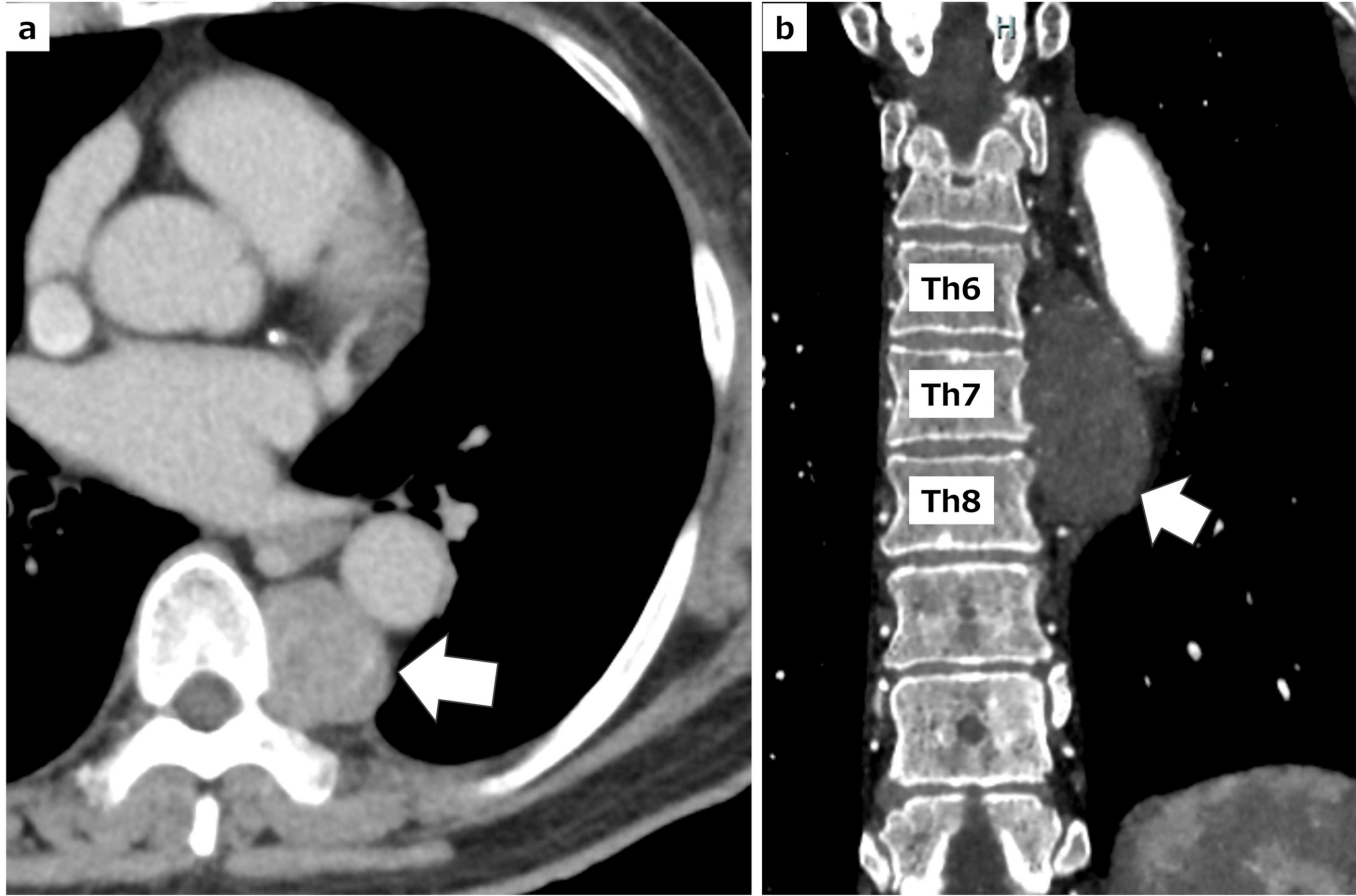
Chest CT findings of the posterior mediastinal tumor a: Axial CT image showing the tumor located between the vertebral bodies and the aorta (white arrow). b: Coronal CT image showing the tumor situated on the left side of the sixth to eighth thoracic vertebrae and adjacent to the descending aorta (white arrow). CT: computed tomography

A 67-year-old man was urgently admitted to the hospital due to a cerebral hemorrhage. Chest computed tomography (CT) scan revealed a posterior mediastinal mass. Following rehabilitation, the patient demonstrated improved functional status and regained the ability to walk, allowing for referral to surgical evaluation. His height was 155 cm, and his weight was 61.5 kg. He had a medical history of bronchial asthma and a smoking history of 20 pack-years. Laboratory tests showed no abnormalities, and pulmonary function tests revealed %VC of 81% and %FEV1 of 51%. Chest CT revealed a tumor measuring 3 × 2.8 × 5 cm, located between the left side of the sixth to eighth thoracic vertebrae and the descending aorta (Figure 1). Magnetic resonance imaging (MRI) of the chest revealed a mass in the posterior mediastinum showing heterogeneous high signal intensity on fat-suppressed T2-weighted imaging and heterogeneous low signal intensity on T1-weighted imaging, accompanied by a split fat sign. These findings were consistent with a diagnosis of schwannoma. We attempted to detect the Adamkiewicz artery using CT angiography but were unable to identify it. Given the tumor's confined location, we determined that enhanced surgical visualization and maneuverability were necessary to minimize complications. Therefore, we planned a robot-assisted thoracoscopic surgery using the da Vinci Xi system with the patient in the prone position.

**Figure 2:**
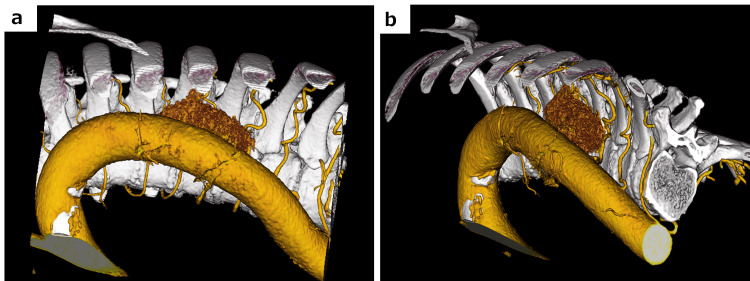
Three-dimensional CT simulation Three-dimensional CT simulation, port placement, and intraoperative findings. a: Simulation of the lateral view from the seventh intercostal space along the inferior axial line. b: Simulation of the lateral view from the ninth intercostal space along the inferior axial line. CT: computed tomography

**Figure 3:**
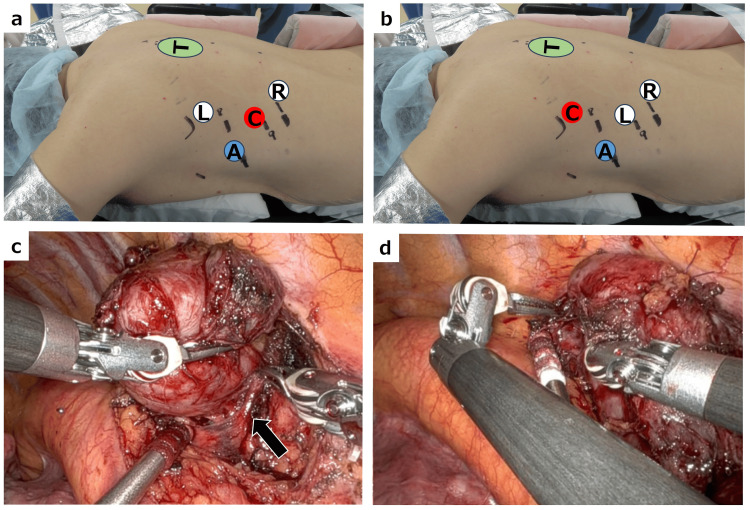
Port placement and intraoperative findings a: Port placement in the prone position. b: Camera replacement to dissect the cranial side of the tumor. c: Intraoperative image showing dissection of the seventh intercostal artery (black arrow) from the tumor surface. d: Intraoperative image showing dissection of the cranial side of the tumor using camera replacement. C: camera (ninth ICS), L: left arm (seventh ICS), R: right arm (10th ICS), A: assist port (eighth ICS), ICS: intercostal space

After the induction of general anesthesia, the patient was positioned prone. An 8-mm da Vinci port was placed in the ninth intercostal space to position the camera, avoiding obstruction of the surgical view by the descending aorta (Figure 2a, 2b). Additional ports were placed in the seventh intercostal space at the posterior axillary line for the left robotic arm and in the 10th intercostal space, 3 cm dorsal to the posterior axillary line. A 12-mm AirSeal port was inserted in the eighth intercostal space at the midaxillary line, and carbon dioxide insufflation was initiated at 6 mmHg. Long bipolar forceps were inserted through the seventh intercostal port, and Maryland forceps were placed in the 10th intercostal port (Figure 3a). The mediastinal pleura overlying the tumor was incised, and dissection was performed outside the tumor capsule to fully expose it. The tumor was found to be contiguous with the sympathetic trunk and was suspected to be a schwannoma of the sympathetic trunk origin. The sympathetic trunk nerve was transected caudally. The tumor was carefully elevated and dissected while monitoring the tumor surface, aorta, and intercostal arteries (Figure 3c). For cephalic dissection, the camera was moved to the seventh intercostal port, and the sympathetic trunk was transected cephalically (Figure 3b, 3d).

The camera was then repositioned in the ninth port, and the tumor was completely excised (Video 1). The blood loss was minimal (<10 mL), the console time was 135 minutes, and the total operative time was 183 minutes. The patient was discharged on postoperative day 4 without complications. Postoperative pain improved rapidly, requiring no analgesics post-discharge, with a maximum Numeric Rating Scale (NRS) score of 2/10. Histological examination confirmed the diagnosis of schwannoma. A follow-up CT performed one year after surgery showed no recurrence, and no late-phase complications were observed.

**Video 1:** Robot-assisted thoracoscopic surgery in the prone position for a posterior mediastinal tumor

## Discussion

We report a case in which a posterior mediastinal schwannoma, located between the aorta and the left side of the sixth to eighth vertebral bodies, was successfully resected using RATS in the prone position. In many cases, schwannomas located in the left thoracic cavity are positioned on the left side of the aorta, making dissection relatively easy. However, in this case, the tumor was situated on the right side of the aorta and located in a space wedged between the vertebral bodies, resulting in increased difficulty during dissection. Although the Adamkiewicz artery could not be detected on CT angiography in this case, the tumor was located at the lower thoracic vertebral level on the left side. Therefore, special attention was required to avoid intercostal artery injury, as it could potentially lead to reduced blood flow in the Adamkiewicz artery and result in paraplegia.

The lateral decubitus position, commonly employed during video-assisted thoracic surgery (VATS) for neurilemmomas, would likely have resulted in the aorta obstructing the deep surgical view. Furthermore, the restricted maneuverability of forceps would have rendered safe dissection even more challenging. Even with a thoracotomy, the aorta would still obstruct the surgical field, and the limited mobility of forceps within the intercostal space would remain problematic. RATS in the prone position provided enhanced visualization and maneuverability, allowing for safe tumor resection while preserving the intercostal artery. Video-assisted thoracoscopic surgery in the prone position for posterior mediastinal tumors was first described in 1995 for patients with dumbbell-shaped tumors [4]. The primary advantage of this approach is that it eliminates the need to reposition the patient to the lateral decubitus position following a spinal procedure. However, its adoption has been limited, possibly due to the technical challenges of thoracoscopic manipulation in the prone position. Since 2021, several reports have emphasized the utility of robot-assisted thoracoscopic surgery in the prone position for resecting dumbbell-shaped tumors [5-7]. The flexibility of the da Vinci system's articulating instruments and magnified vision has been a key factor in the successful implementation of this technique. Although the tumor in our case was not dumbbell-shaped, its confined location posed significant challenges due to the risk of intercostal arterial injury. By adopting the prone position, the obstruction of the surgical view caused by the aorta could be avoided. This position facilitated obtaining an improved visualization by allowing the lung to collapse under the effects of gravity and insufflation with carbon dioxide. Moreover, reducing an arm for lung retraction helped minimize interference during the procedure. Notably, we placed the da Vinci ports in the seventh, ninth, and 10th intercostal spaces, which were positioned lower than in previous reports [5-7]. This port placement strategy was designed to avoid obstruction of the surgical view by the lung and aorta while allowing simultaneous monitoring of the tumor, aorta, and intercostal arteries. Although this configuration optimized the view of the tumor's caudal side, visualizing its cephalic aspect remained challenging. The relocation of the camera port to the most cranial port was beneficial for this purpose.

## Conclusions

This case highlights the feasibility and safety of robot-assisted thoracoscopic surgery in the prone position for the resection of posterior mediastinal tumors, particularly those in confined anatomical locations. This approach offers superior visualization and maneuverability, reducing the risk of intercostal artery injury and potential complications related to the Adamkiewicz artery.
